# Exploring Neural Correlates between Anxiety and Inhibitory Ability: Evidence from Task-Based fNIRS

**DOI:** 10.1155/2024/8680134

**Published:** 2024-06-27

**Authors:** Difan Wang, Bingyan Lin, Ying Huang, Zh Yeng Chong, Jiaxue Du, Qin Yuan, Yinmayue Tang, Qiming Xu, Wei Xu

**Affiliations:** ^1^Department of Internal Medicine, Psychological Counseling and Service Center, Graduate School of Medical College of Chinese PLA General Hospital, Beijing, China; ^2^Faculty of Psychology, Beijing Normal University, Beijing 100875, China; ^3^Laboratory of Awareness Brain Science, Beijing, China; ^4^Department of Clinical Psychology, School of Health in Social Science, University of Edinburgh, Edinburgh, Scotland, UK; ^5^School of Psychology, Central China Normal University, Wuhan, China; ^6^Department of Social and Behavioral Sciences, City University of Hong Kong, Hong Kong, China; ^7^Department of Psychology, Lingnan University, Hong Kong, China

## Abstract

**Background:**

Cognitive control impairments in anxiety disorders are thought to be associated with deficiencies in the prefrontal network. However, a precise neural explanation for these impairments is still lacking. This study seeks to compare inhibitory ability between individuals with anxiety and healthy controls, as well as to explore the neural correlates of anxiety-related inhibitory deficits within a clinical context.

**Materials and Methods:**

A total of 118 participants were recruited, including 59 patients with anxiety and 59 matched healthy controls (HCs). Anxiety and inhibitory ability were evaluated using Zung's Self-rating Anxiety Scale (SAS), the color word Stroop task, and verbal fluency task (VFT). Additionally, changes in oxyhemoglobin (Oxy-Hb) concentrations were measured using functional near-infrared spectroscopy (fNIRS).

**Results:**

Compared to HCs, the anxiety group exhibited reduced cortical activation in prefrontal cortex (PFC) channels, prolonged inhibitory speed and lower inhibitory accuracy during Stroop task, and diminished VFT performance (all *p* < 0.05). Significant negative correlations were observed between SAS scores and inhibitory ability, as well as with PFC activation. Conversely, PFC activation showed positive correlations with inhibitory ability. Importantly, activation in the dorsolateral PFC during VFT partially mediated the association between anxiety and inhibitory performance.

**Conclusions:**

This study reveals neural characteristics associated with inhibitory abilities in anxiety disorders and identifies neural correlations between anxiety and inhibitory performance. These findings illuminate the impact of anxiety on inhibitory abilities and propose intervention targets to enhance these abilities in individuals with anxiety disorders, thereby suggesting more effective therapeutic strategies.

## 1. Introduction

Anxiety disorders are recognized as the most prevalent psychological disorders globally [1, 2, 3]. Despite this, the fundamental principles and neural characteristics of anxiety remain incompletely understood [4]. Moreover, a significant proportion of individuals with anxiety disorders-ranging from 71% to 97.8% are not accurately diagnosed [5], and approximately 41% do not receive treatment [6]. This exacerbates the severity of the condition. Gaining a deeper understanding of the mechanisms and characteristics of anxiety disorders may lead to improvements in diagnosis and treatment, thereby contributing to more rapid and lasting amelioration of symptoms. Such advancements could also alleviate the considerable burden that high anxiety rates place on healthcare systems and economies worldwide [7, 8].

Anxiety can significantly impact cognitive performance, with attentional control theory providing insight into this relationship. According to this theory, anxiety negatively adverse core executive functions of attentional control, including inhibition and shifting [9]. Research indicates that individuals with anxiety-related disorders commonly exhibit deficits in inhibitory functions [10], and those predisposed to anxiety are more likely to experience impairments in cognitive inhibition, especially when processing resources are limited [11]. Anxiety often involves a perceived loss of control and difficulty regulating emotions, leading to diminished attentional focus [4]. In conditions like Generalized Anxiety Disorder (GAD), the inability to control worry is linked to impaired cognitive inhibition, a critical subprocess of cognitive control. This impairment correlates with the severity and outcomes of the disorder [12]. ERP studies have shown that highly anxious individuals may experience delayed responses during inhibition tasks and reduced brain activity in specific regions associated with inhibitory processes [13]. Understanding how anxiety affects inhibitory functions could improve the diagnosis and treatment of anxiety disorders, potentially offering faster and more effective relief.

Despite advances in previous research that have improved our understanding of the neural mechanisms involved in anxiety and inhibition, studies have yielded mixed results. For instance, some research reports stronger activation of a cognitive control network, including the dorsolateral PFC (DLPFC), in anxious subjects during trial-to-trial adjustments of cognitive control tasks [14], while others indicate weaker activation of the left DLPFC in anxious subjects in distracter inhibition paradigms [15]. Moreover, diminished DLPFC activation has been noted during tasks requiring emotional interference inhibition in individuals with Generalized Anxiety Disorder (GAD) [16], and untreated GAD patients have shown increased perfusion in specific brain regions during verbal fluency tasks (VFTs) [17]. These inconsistent findings may be due to variations in several factors, including the choice of behavioral paradigms and the methods employed for statistical comparisons. Additionally, the findings of an ERP study demonstrated that individuals with low social anxiety tend to prioritize proactive control processes, which are driven by DLPFC activity. Conversely, individuals with high social anxiety exhibit a preference for reactive control processes, implicating the dorsal anterior cingulate cortex [18]. Furthermore, variations in trait anxiety appear to influence the relationship between prefrontal cortex (PFC) glutamate levels and DLPFC activation, ultimately impacting cognitive control task performance [19]. In summary, the neural correlates underlying this relationship are complex and remain incompletely understood, requiring further research efforts.

This study specifically focuses on exploring the neural links between anxiety and inhibitory control, which involves the ability to suppress responses to irrelevant information while maintaining focus on a cognitive goal [20]. The color-word Stroop task is widely recognized as a standard method for evaluating inhibitory control, response inhibition, cognitive flexibility, executive functioning, information processing speed, working memory, and selective attention [21, 22, 23, 24]. It relies on response inhibition, generating conflict between two simple options of the same stimulus feature [21], requiring individuals to inhibit one behavior, such as reading a word, in favor of another, like naming the color [25], thereby providing an efficient tool for assessing inhibitory ability.

While inhibitory control is commonly evaluated through the Stroop task, research suggests that the VFT can effectively assess individual inhibitory control [26, 27, 28]. This is because VFT relies on executive functioning skills, encompassing general self-regulation and control processes involving monitoring and inhibitory control [29]. Specifically, during word production in VFT, individuals must inhibit inappropriate responses and semantically related words, engage in self-monitoring, and avoid perseverations [30]. VFT demands efficient organization of verbal retrieval and recall, along with cognitive self-monitoring (participants need to keep track of previous responses), deliberate self-initiation, and inhibition of responses when necessary [26, 31]. This demands participants to employ a strategy not typically used in everyday communication, necessitating the suppression of automatically activated semantic neighbors for the listed words during the task [32, 33]. Additionally, switching, a key cognitive strategy for evoking words in VFT [34, 35], has been linked to inhibitory control and cognitive flexibility [35, 36, 37]. Moreover, previous research has utilized VFT to measure inhibitory-related abilities. For instance, Carpenter et al. [38] employed VFT to measure cognitive control, as adjusting cognitive control demands is essential for successful lexical retrieval in VFT, with response inhibition playing a crucial role in suppressing irrelevant words or impulses [38]. Additionally, Patra et al. [39] observed an association between performances on the verbal fluency and Stroop tasks, thus implicating the role of inhibition in verbal fluency performance.

Furthermore, VFT is a valuable tool for investigating hemodynamic responses in functional near-infrared spectroscopy (fNIRS) scans. It offers simplicity, requires minimal resources, and is commonly employed to induce PFC activation in fNIRS studies [40, 41, 42, 43, 44, 45]. fNIRS, as a noninvasive and safe neuroimaging technique, provides superior spatial resolution compared to electroencephalogram (EEG) and improved temporal resolution relative to functional magnetic resonance imaging (fMRI) [46]. Moreover, fNIRS offers enhanced tolerance to motion artifacts and is notable for its cost-effectiveness, portability, and ease of use compared to other brain imaging technologies [47]. fNIRS-VFT has proven effective in assessing brain function in psychiatric disorders such as anxiety disorders [48, 49, 50, 51]. Hence, in addition to the conventional Stroop task, by leveraging the advantages of fNIRS-VFT and the potential of VFT in assessing inhibitory control, this study utilizes fNIRS-VFT to explore the neural correlation between anxiety and inhibitory control.

To fully investigate the relationship between anxiety and inhibition, this study aims to (1) compare anxiety and healthy control subjects and (2) examine whether the brain activation of VFT mediates the relationship between anxiety and inhibitory performance. To the best of our knowledge, this study represents the first exploration of this potential role. The hypotheses are as follows: Hypothesis 1: Individuals with anxiety disorders exhibit lower levels of prefrontal activation and inhibition deficits compared to healthy controls. Hypothesis 2: The severity of anxiety correlates with prefrontal cortex activation during the VFT and with inhibition levels. Hypothesis 3: Prefrontal activation during VFT mediates the relationship between anxiety and inhibitory performance.

## 2. Materials and Methods

### 2.1. Participants and Procedures

This study recruited a total of 118 participants, comprising 59 individuals diagnosed with anxiety disorders (including 42 with generalized anxiety disorder, 15 with panic disorder, and 2 with social anxiety disorder), as well as 59 healthy controls (HCs). Participants ranged in age from 19 to 65 years. Recruitment was conducted through posters and online advertisements at the Chinese PLA General Hospital and in the local community. The inclusion criteria for the anxiety group were (1) a professional diagnosis of anxiety disorders by psychiatrists based on the Diagnostic and Statistical Manual of Mental Disorders, Fifth Edition (DSM-5) [52]; (2) a score above 50 on the Self-rating Anxiety Scale (SAS), indicating at least mild anxiety [53]; (3) aged 19 or older; (4) right-handedness; (5) signed informed consent. Exclusion criteria included visual impairments, psychological disorders other than anxiety, and brain injuries significantly impacting cognitive function.

Healthy controls were individuals with SAS scores below 50, indicating the absence of an anxious state [53]. They were screened through a brief face-to-face interview by psychiatrists to ensure the absence of significant psychological disorders. The anxiety and HC groups were matched for age, right-hand dominance, and gender. All participants signed informed consent had normal vision, no history of attention deficit or dyslexia, and were native Chinese speakers.

The anxiety group consisted of 25 males and 34 females (mean age = 35.85 years; SD = 6.42), while the healthy control group included 23 males and 36 females (mean age = 36.51 years; SD = 6.03). Upon enrollment, participants underwent assessments involving questionnaires, the color word Stroop task, and fNIRS measurements, administered by trained doctoral-level interviewers. These assessments were designed to evaluate anxiety severity, inhibition, and cortical activation, respectively.

The study is part of a larger interventional study (preregistered at https://www.chictr.org.cn/; registration number: ChiCTR2300071053) and received approval from the Institutional Review Board of the Medical Ethics Committee of Chinese PLA General Hospital (approval no. of Ethics Committee: S2023-120-02). All procedures conformed to the ethical standards of relevant national and institutional committees on human experimentation and the Declaration of Helsinki in 1964.

### 2.2. Measures

#### 2.2.1. Anxiety

Zung's Self-rating Anxiety Scale (SAS), a norm-referenced scale, is widely utilized as a screener for anxiety disorders [53]. The scale comprises 20 items that reflect subjective feelings of anxiety, scored on a 4-level scale. The raw total score is multiplied by 1.25 to obtain the standard score. A standard below 49 is considered normal, 50–59 indicates mild anxiety, 60–69 suggests moderate anxiety, and scores above 69 are indicative of severe anxiety. In this study, the Cronbach's alpha coefficient for the SAS was 0.97.

#### 2.2.2. The Color-Word Stroop Task

**Figure 1:**
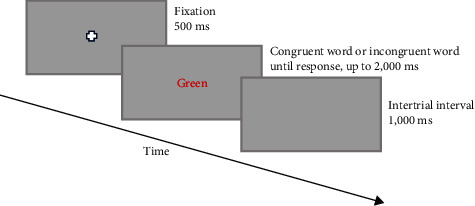
The stimulus display sequence is shown for an incongruent Stroop trial.

The color-word Stroop task involves presenting color names displayed in incongruent colors, which creates a Stroop interference effect. Using PsychoPy, the task consisted of 240 trials divided equally between congruent and incongruent conditions (Figure 1). In the incongruent condition, the displayed color of the word did not match its name (e.g., the word green “green” displayed in red). Words were displayed on a gray computer screen positioned 40 cm from the participants. Stimuli comprised random presentation of four-color names (red, green, yellow, or blue) in one of these colors, with no consecutive repetition of color names or presentation colors. Participants pressed keys corresponding to the color of the word (“D,” “F,” “J,” or “K” for “red,” “yellow,” “blue,” or “green,” respectively). Accuracy and average reaction time on response-correct trials were extracted. Interference was calculated by subtracting the reaction time on congruent trials from incongruent trials [24]. Sixteen practice words preceded the actual task for participant familiarization.

#### 2.2.3. Verbal Fluency Task

Participants performed a Chinese version of the VFT during the daytime. The task included a 30-s pretask baseline, a 60-s task period, and a 60-s post-task baseline. During both baseline periods, participants were instructed to repetitively count from 1 to 5. In the task period, participants generated as many phrases as possible using three commonly used Chinese characters, such as “白” (white), “北” (north), and “大” (big). The three given characters changed every 20 s to minimize silent periods.

#### 2.2.4. fNIRS Measurement

**Figure 2:**
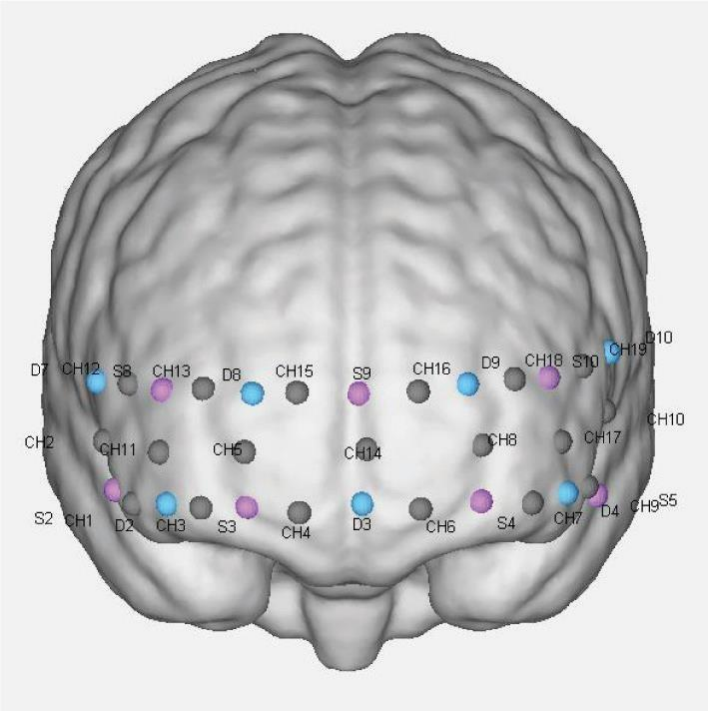
The position of SD and channels.

Participants were seated in a quiet room, and a multichannel continuous-wave near-infrared spectrometer (NirSmart-6000A, Danyang Huichuang Medical Equipment Co., Ltd., China) was used to record changes in concentrations of oxygenated hemoglobin (HbO) and deoxyhemoglobin (HbR) during the VFT. The neurovascular coupling mechanism was leveraged to infer brain activity from these changes. The system, comprising LED light sources and avalanche photodiodes (APD) as detectors at wavelengths of 730 and 850 nm and a sampling rate of 11 Hz, was employed. Using the FPz channel (10/20 international system) as the center of the middle probe, a total of 14 source-detector (SD) probes with a fixed 3-cm interprobe distance were arranged to cover each participant's bilateral prefrontal and temporal cortices, with the lowest probes along the Fp1-Fp2 line. This configuration established a total of 19 NIRS channels (Figure 2), with the channels and corresponding brain regions presented in Table S1.

### 2.3. Data Processing and Analysis

Statistical analysis was conducted using R software, the NirSpark software package, and SPSS version 25. R software was utilized for pre-processing data from the color word Stroop task, while NirSpark software (Danyang Huichuang Medical Equipment Co., Ltd., China) analyzed fNIRS data. Preprocessing involved motion artifacts correction using moving SD and cubic spline interpolation, along with a low-pass filtering of 0.20 Hz to remove physiological noise (e.g., respiration, cardiac activity) [54]. The modified Beer–Lambert law converted optical densities into changes in Oxy-Hb and Deoxy-Hb concentrations. Oxy-Hb, with its superior signal-to-noise ratio [55], was the primary focus. VFT block waveforms were calculated with a block range set of 0–125 s, a prebaseline range set of 0–10 s, and a postbaseline range set of 70–125 s. We used a 60 s task period of constructing phrases as the time window to analyze mean Oxy-Hb changes. Linear fitting was applied to the data between these two baselines.

Group differences in demographic and fNIRS data were assessed using chi-square tests or independent samples *t*-tests. A mixed ANOVA with the dependent variables reaction time (RT) and accuracy in the two conditions of the Stroop task (congruency, incongruency) was performed. The grouping variable of anxiety group and HCs was included as a between-subject variable. Normality of the data was confirmed using the Shapiro–Wilk test. Pearson correlation analysis was conducted to investigate associations among anxiety, brain activation, and inhibitory ability. Statistical significance was adjusted for multiple comparisons using the false discovery rate (FDR). Mediation models were tested using Process 3.5 [56] with a bias-corrected nonparametric percentile bootstrap test. Anxiety is the independent variable, inhibitory performance is the dependent variable, and cortical activation is the mediator variable. Both direct and indirect effects were assessed, using bootstrapping with 5,000 resamples and 95% confidence intervals, fully adjusted for covariates. The indirect effect was deemed significant if 0 was not included in the confidence interval.

## 3. Results

### 3.1. Demographics, Clinical Variables, and Inhibitory Performances

The demographic characteristics, clinical variables, and inhibitiory performances of participants in each group are presented in Table 1. There is no statistical difference between the two groups in age (*t* = −0.58, *p* = 0.57) and gender (chi-square = 0.14, *p* = 0.71). In contrast, the mean SAS scores of participants with anxiety were significantly higher than HCs (*t* = 23.88, *p*  <  0.001). Additionally, the VFT performance of the anxiety group was significantly lower than that of HCs (*t* = −10.18, *p*  <  0.001).

Mauchly's test confirmed the assumption of sphericity for conducting ANOVA (*p* > 0.999). Examining reaction time, mixed ANOVA revealed a significant main effect of condition (*F* (1, 116) = 308.25, *p* < 0.001, *ηp^2^* = 0.112) and anxiety group (*F* (1, 116) = 172.25, *p* < 0.001, *ηp^2^* = 0.586). An interaction effect between condition and group was also significant (*F* (1, 116) = 37.01, *p* < 0.001, *ηp^2^* = 0.015), indicating a larger RT change between congruent and incongruent conditions in the anxiety group. Post hoc analyses confirmed significantly longer RT for the anxiety group across all conditions (*p* < 0.001; Table S2 and Figure S1). Turning to accuracy, Mixed ANOVA revealed a significant main effect of condition (*F* (1, 116) = 26.78, *p* < 0.001, *ηp^2^* = 0.007), with greater accuracy during the congruent condition for all participants. A significant main effect of the group was observed (*F* (1, 116) = 10.97, *p* < 0.001, *ηp^2^* = 0.084). The interaction effect between condition and group was significant (*F* (1, 116) = 17.31 *p* < 0.001, *ηp^2^* = 0.005), indicating a larger accuracy change between congruent and incongruent conditions in the anxiety group. Post hoc analyses with Bonferroni corrections revealed lower accuracy for the anxiety group compared to HCs in the incongruent condition (*p* < 0.05), though it was not significant in the congruent condition (*p*=0.06; Table S2 and Figure S1).

### 3.2. Mean Oxy-Hb Changes during VFT between Two Groups

**Figure 3:**
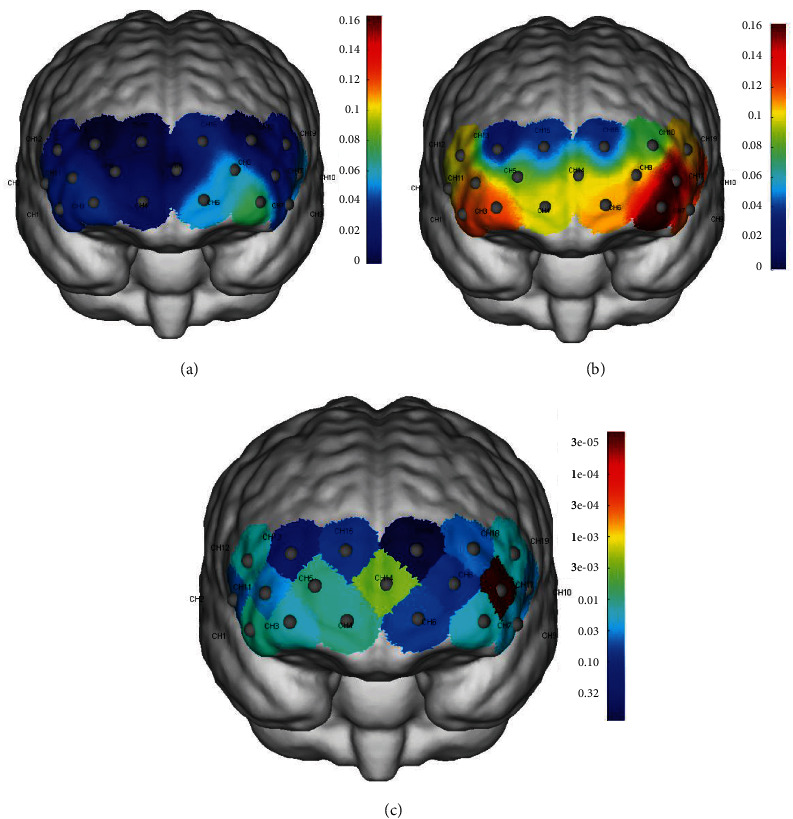
(a) Mean Oxy-Hb changes during the VFT of patients group. (b) Mean Oxy-Hb changes during the VFT of HCs. (c) *p*-Values of each channel activation for patients compared to HCs.

During the VFT task period, the anxiety group showed less cortical activation in the hemodynamic responses of Oxy-Hb than HCs at Channels 1, 7, 11, 17, and 18 (mainly located in the DLPFC), Channels 2, 10, 12, and 19 (mainly located in the pars triangularis Broca's area), Channels 3 and 4 (mainly located in the orbitofrontal area), Channels 5 and 14 (mainly located in the frontopolar area), Channel 9 (mainly located in the Inferior prefrontal gyrus) (FDR corrected *p* < 0.05; Figures 3(a), 3(b), and 3(c) and Table 2). Deoxy-Hb with a lower signal-to-noise ratio than Oxy-Hb was not our primary indicator, therefore, its results are presented in Table S3.

### 3.3. Functional Connectivity between Two Groups

**Figure 4:**
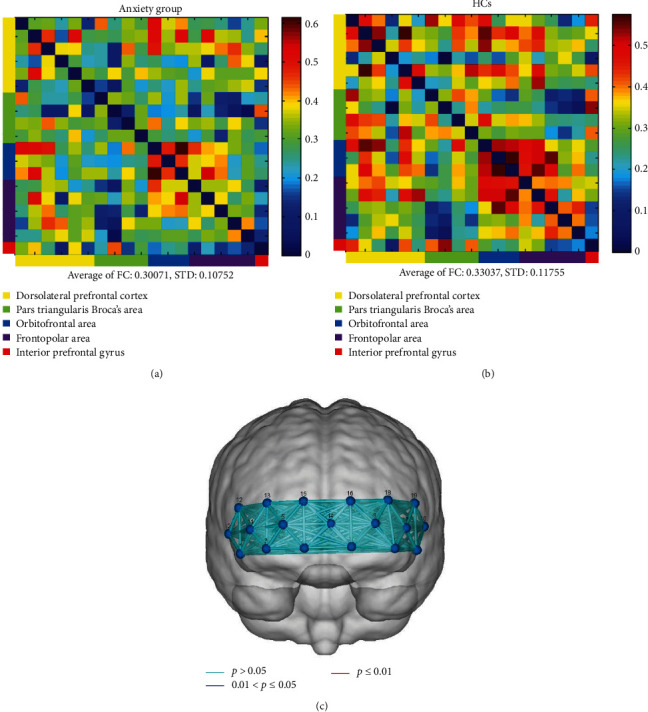
(a, b) Connectivity between the hemodynamic responses of 19 channels. (c) Pair comparisons between two groups.

After functional connectivity calculation, two 19 × 19 correlation matrices were generated for anxiety group and HCs (Figures 4(a) and 4(b)). The mean channel-to-channel connectivity strength was 0.33 (SD = 0.12) for HCs and 0.30 (SD = 0.11) for participants with anxiety. There was no significant difference between the two groups (*t* = −0.76, *p*=0.45) (Figure 4(c)).

### 3.4. Correlations between Anxiety, Inhibitory Abilities, and Mean Oxy-Hb Changes

Pearson correlation analysis revealed that SAS scores negatively correlated with VFT performance (*r* = −0.78, *p* < 0.001) and mean Oxy-Hb changes in various PFC regions, including the DLPFC (Channel 1 (*r* = −0.31, *p* < 0.01), Channel 7 (*r* = −0.26, *p* < 0.01), Channel 11 (*r* = −0.33, *p* < 0.001), Channel 17 (*r* = −0.47, *p* < 0.001), and Channel 18 (*r* = −0.25, *p* < 0.001)), pars triangularis Broca's area (Channel 2 (*r* = −0.35, *p* < 0.001), Channel 10 (*r* = −0.22, *p* < 0.05), Channel 12 (*r* = −0.29, *p* < 0.01), and Channel 19 (*r* = −0.23, *p* < 0.05)), orbitofrontal area (Channel 3 (*r* = −0.24, *p* < 0.05) and Channel 4 (*r* = −0.25, *p* < 0.05)), frontopolar area (Channel 5 (*r* = −0.34, *p* < 0.001), Channel 8 (*r* = −0.20, *p* < 0.05), Channel 15 (*r* = −0.51, *p* < 0.001), and Channel 16 (*r* = −0.26, *p* < 0.01)), and inferior prefrontal gyrus (Channel 9 (*r* = −0.23, *p* < 0.05)). Conversely, SAS scores were positively correlated with the difference score in Stroop speed (*r* = 0.73, *p* < 0.001) and accuracy (*r* = 0.46, *p* < 0.001).

Furthermore, significant positive correlations were observed between VFT performance and mean Oxy-Hb changes in various PFC regions, including the DLPFC (Channel 1 (*r* = 0.30, *p* < 0.01), Channel 7 (*r* = 0.25, *p* < 0.05), Channel 11 (*r* = 0.31, *p* < 0.01), Channel 13 (*r* = 0.27, *p* < 0.01), Channel 17 (*r* = 0.50, *p* < 0.001), and Channel 18 (*r* = 0.23, *p* < 0.05)), pars triangularis Broca's area (Channel 2 (*r* = 0.28, *p* < 0.01), Channel 10 (*r* = 0.19, *p* < 0.05), Channel 12 (*r* = 0.23, *p* < 0.05), and Channel 19 (*r* = 0.23, *p* < 0.05)), orbitofrontal area (Channel 3 (*r* = 0.21, *p* < 0.05)), frontopolar area (Channel 5 (*r* = 0.29, *p* < 0.01), Channel 8 (*r* = 0.21, *p* < 0.05), Channel 15 (*r* = 0.46, *p* < 0.001), and Channel 16 (*r* = 0.22, *p* < 0.05)), and inferior prefrontal gyrus (Channel 9 (*r* = 0.23, *p* < 0.05)).

Moreover, the difference score in Stroop speed negatively correlated with VFT performance (*r* = - 0.59, *p* < 0.001), as well as with the mean Oxy-Hb changes in the DLPFC (Channel 1 (*r* = −0.26, *p* < 0.01), Channel 7 (*r* = −0.24, *p* < 0.05), Channel 11 (*r* = −0.40, *p* < 0.001), Channel 17 (*r* = −0.68, *p* < 0.001), and Channel 18 (*r* = −0.36, *p* < 0.001)), pars triangularis Broca's area (Channel 2 (*r* = −0.39, *p* < 0.001), Channel 10 (*r* = −0.28, *p* < 0.01), Channel 12 (*r* = −0.31, *p* < 0.01), and Channel 19 (*r* = −0.26, *p* < 0.01)), orbitofrontal area (Channel 3 (*r* = −0.21, *p* < 0.05), Channel 4 (*r* = −0.29, *p* < 0.01), and Channel 6 (*r* = −0.23, *p* < 0.05)), frontopolar area (Channel 5 (*r* = −0.36, *p* < 0.001), Channel 8 (*r* = −0.24, *p* < 0.05), Channel 15 (*r* = −0.45, *p* < 0.001), and Channel 16 (*r* = −0.34, *p* < 0.001)), and inferior prefrontal gyrus (Channel 9 (*r* = −0.21, *p* < 0.05)). Additionally, there are negative correlations between the difference scores in Stroop accuracy and mean Oxy-Hb changes in the DLPFC (Channel 13 (*r* = −0.21, *p* < 0.05) and Channel 17 (*r* = −0.26, *p* < 0.01)), frontopolar area (Channel 16 (*r* = −0.22, *p* < 0.05)), as well as with VFT performance (*r* = −0.35, *p* < 0.001). Correlation results of all studied variables are presented in Table S4, with all *p*-values adjusted using false discovery rate correction.

### 3.5. The Mediating Role of Mean Oxy-Hb Changes between Anxiety and Inhibitory Abilities

**Figure 5:**
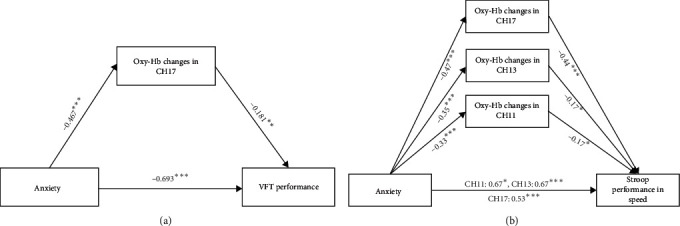
(a, b) Results of mediation analysis. CH = channel; VFT = verbal fluency task; Stroop performance in speed = difference time between incongruent and congruent condition. *⁣*^*∗*^*p* < 0.05, *⁣*^*∗∗*^*p* < 0.01, *⁣*^*∗∗∗*^*p* < 0.001.

We identified a significant mediating role of mean Oxy-Hb changes in Channel 17, primarily located in the DLPFC, in the relationship between anxiety and VFT performance (Figure 5(a)). Specifically, anxiety scores were found to negatively predict Oxy-Hb changes in Channel 17 (*β* = −0.467, *p* < 0.001) and VFT performance (*β* = −0.693, *p* < 0.001), while activation in channel 17 positively predicted VFT performance (*β* = 0.181, *p* < 0.01). The bootstrap analysis with 5,000 bootstrap samples showed the total effect is −0.1001 (SE = 0.0075; 95% CI: −0.1150, −0.0852). The direct effect is −0.0892 (SE = 0.0083; 95% CI: −0.1056, −0.0729). For the indirect effect, 95% bootstrap confidence intervals (CIs) without “zero” indicate the significant mediation effect. The mediating effect is −0.0109 (SE = 0.0047; 95% CI: −0.0205, −0.0023).

Furthermore, we observed a significant mediating role of mean Oxy-Hb changes in Channels 11, 13, 17 (mainly located in the DLPFC) in the relationship between anxiety and Stroop performance in speed (Figure 5(b)). Specifically, the anxiety scores negatively predicted Oxy-Hb changes in Channel 11 (*β* = −0.33, *p* < 0.001), channel 13 (*β* = −0.35, *p* < 0.001), and channel 17 (*β* = −0.47, *p* < 0.001). Simultaneously, anxiety scores positively predicted speed difference during Stroop in channel 11 (*β* = 0.67, *p* < 0.05), channel 13 (*β* = 0.67, *p* < 0.001), and channel 17 (*β* = 0.53, *p* < 0.001). Furthermore, channel activation in channel 11 (*β* = −0.17, *p* < 0.05), channel 13 (*β* = −0.17, *p* < 0.05), and channel 17 (*β* = −0.44, *p* < 0.001) negatively predicted speed difference. The total effect is 0.0027 (SE = 0.0002; 95% CI: 0.0022, 0.0032). The direct effect ranged from 0.0019 to 0.0025 (channel 11 : 0.0025 (SE = 0.0004; 95% CI: 0.0080, 0.0098), channel 13 : 0.0025 (SE = 0.0002; 95% CI: 0.0020, 0.0029), and channel 17 : 0.0019 (SE = 0.0002; 95% CI: 0.0015, 0.0024)). The mediating effect ranged from 0.0002 to 0.0003 (channel 11 : 0.0008 (SE = 0.0001; 95% CI: 0.0001, 0.0004), channel 13 : 0.0002 (SE = 0.0001; 95% CI: 0.0001, 0.0004), and channel 17 : 0.0008 (SE = 0.0002; 95% CI: 0.0003, 0.0012)). However, this mediating effect was not observed in the case of Stroop performance in accuracy.

## 4. Discussion

This study is pioneering in exploring the potential mediation role of cortical activation between anxiety and inhibitory ability using fNIRS. Our findings indicate a negative correlation between anxiety and inhibitory ability, as well as cortical activation in the PFC area during VFT. Conversely, PFC activation showed positive correlations with inhibitory ability. Notably, the activation in the DLPFC area partially mediated the relationship between anxiety and inhibitory performance. Regarding the group differences, the anxiety group exhibited abnormal activation patterns and diminished inhibitory ability than HCs. Specifically, individuals with anxiety demonstrated significantly lower cortical activation in the hemodynamic responses of Oxy-Hb mainly in the PFC channels than HCs. Additionally, the anxiety group demonstrated prolonged inhibitory speed and lower inhibitory accuracy during the Stroop task, as well as diminished VFT performance. However, no differences in functional connectivity during the VFT task were observed between the two groups.

The study found that individuals with anxiety exhibited significantly less cortical activation in the hemodynamic responses of Oxy-Hb in the PFC channels compared to HCs, aligning with previous studies [57, 58]. Further investigation demonstrated a negative association between anxiety severity and Oxy-Hb changes in PFC, consistent with earlier studies linking anxiety to reduced prefrontal activation [15, 59, 60]. This may be attributed to anxiety directly impacting the prefrontal cortex, affecting functions such as working memory and cognitive executive processes [61]. While prior research suggested a potential difference in functional connectivity between individuals with social anxiety disorders and healthy controls during unattended neutral face tasks [62], our study revealed no differences in functional connectivity during the VFT between the two groups. This discrepancy in results could be attributed to the use of different tasks in our study. Additionally, changes in prefrontal cortex activation have been reported as a neural signature of anxiety [63]. These collective findings underscore the integral role of the PFC in anxiety and highlight its potential as a target for adjunct diagnosis and treatment efforts, specifically offering valuable insights into using fNIRS to assess PFC activation as an adjunct diagnostic tool for anxiety disorders.

Our findings reveal that anxiety patients exhibit reduced inhibitory ability compared to healthy controls. This observation is consistent with prior research [64, 65] and supports the attentional control theory [9], which posits that anxiety impairs executive attentional control, including inhibition. Further investigation demonstrated an association between higher anxiety levels and diminished inhibitory performance, echoing findings from previous studies [66, 67]. Anxiety, often accompanied by unpleasant feelings such as worry [68], may hampers executive control, making it difficult for individuals to cease worrying, consequently impacting cognitive processes [69]. While earlier studies with older adults suggested that mild anxiety might enhance cognitive performance [70, 71], these findings are specific to elderly populations and may not be generalizable. Moreover, they imply that only mild anxiety correlates with improved cognitive performance, while more severe anxiety is associated with reduced cognitive abilities. Overall, our findings contribute to the growing body regarding the connection between anxiety and inhibition, emphasizing the need for further investigation, particularly considering potential variations across diverse populations.

This study also found positive correlations between PFC activation during VFT and inhibitory performance, linking inhibition to prefrontal brain regions [72, 73]. Connectivity between the DLPFC and the inferior frontal gyrus may facilitate inhibitory processes, reflecting cognitive control demands [74]. Our results contribute to the burgeoning research suggesting that PFC cortical activation could serve as a biomarker for inhibitory ability.

According to our study's mediation model, activation of the DLPFC during the VFT task mediates the relationship between anxiety and inhibitory performance. This finding is supported by prior research indicating that anxiety compromises the functioning of the left DLPFC [75, 76]. As the DLPFC is located in the middle frontal gyrus, it is integral to cognitive functioning and notably implicated in psychiatric disorders [77, 78]. The DLPFC plays a crucial role in inhibition, cognitive flexibility, decision-making, working memory, response selection, and emotion regulation, particularly in anxiety [79, 80, 81]. Furthermore, previous research emphasizes the role of DLPFC in cognitive control [82] and highlights its altered activation in individuals with high trait anxiety during tasks requiring cognitive control [15, 83, 84]. This finding shed light on how DLPFC activation serves as a crucial link connecting anxiety to inhibitory performance. It contributes to a deeper understanding of how anxiety influences inhibitory ability and suggests potential intervention targets for improving outcomes. Interventions targeting DLPFC activity may enhance inhibitory abilities in individuals with anxiety disorders and, consequently, improve their behavioral performance. This advancement in knowledge regarding the neural basis of anxiety disorders provides valuable insights for the development of more effective treatment strategies.

This research has several limitations that warrant attention in future studies. First, although the VFT is one of the most used paradigms to assess cognitive functioning in fNIRS psychiatric research, and some evidence supports its involvement in inhibitory control, it is also viewed by many researchers merely as a paradigm for activating the PFC. Therefore, further research is needed to further verify whether VFT genuinely characterizes inhibitory control. Second, the temporal separation between anxiety assessments, fNIRS measurements, and cognitive tasks aimed to explore the dynamic interplay between enduring anxiety states and real-time cognitive processes. While this design choice provides valuable insights into potential carryover effects, we acknowledge its impact on the interpretation of our findings. Encouragingly, future research could delve into the relationship between anxiety, neuro processes, and cognitive processes in more tightly coupled time frames, fostering a more nuanced understanding of these dynamics. Finally, the spatial resolution of fNIRS is inferior to that of fMRI. However, most of our findings align with previous studies observed through different neuroimaging modalities, future studies should combine both fNIRS and fMRI to validate these findings.

## 5. Conclusions

In summary, our study investigated differences in inhibitory ability and brain activation patterns between patients with anxiety disorders and healthy controls, while also exploring the underlying links between anxiety and inhibitory ability. These findings propose potential enhancements to the diagnostic process for anxiety disorders by incorporating fNIRS or behavioral task indicators, thus paving the way for more comprehensive diagnostic approaches. Moreover, our mediation model sheds light on how anxiety impacts inhibitory abilities and suggests intervention targets to enhance these abilities in individuals with anxiety disorders, ultimately improving their behavioral performance. This contributes to advancing our understanding of the neural mechanisms underlying anxiety disorders and guiding the development of more effective treatment strategies.

## Figures and Tables

**Table 1:** Demographic, clinical data, and inhibition performance (M ± SD).

Variables	Anxiety group (*n* = 59)	HCs (*n* = 59)	*t/χ* ^2^	*p*
Age	35.85 ± 6.42	36.51 ± 6.03	−0.58	0.57
Gender	—	—	0.14	0.71
Male	25 (42.4%)	23 (39%)	—	—
Female	34 (57.6%)	36 (61%)	—	—
SAS score	67.42 ± 10.22	33.77 ± 3.56	23.88	<0.001
VFT performance	6.12 ± 1.61	9.37 ± 1.86	−10.18	<0.001

*Note*. SAS = Zung's Self-Rating Anxiety Scale.

**Table 2:** Channel activation between groups.

CH	S–D	*t*	*p*
1	S2–D2	−3.024	<0.05
2	S2–D7	−2.224	<0.05
3	S3–D2	−2.882	<0.05
4	S3–D3	−3.168	<0.05
5	S3–D8	−3.132	<0.05
6	S4–D3	−1.972	0.065
7	S4–D4	−2.747	<0.05
8	S4–D9	−1.861	0.074
9	S5–D4	−2.549	<0.05
10	S5–D10	−2.365	<0.05
11	S8–D2	−2.426	<0.05
12	S8–D7	−2.812	<0.05
13	S8–D8	−1.279	0.215
14	S9–D3	−3.709	<0.01
15	S9–D8	−1.854	0.074
16	S9–D9	−0.151	0.880
17	S10–D4	−5.134	<0.001
18	S10–D9	−2.178	<0.05
19	S10–D10	−2.696	<0.05

*Note*. S = source; D = detector; CH = channel.

## Data Availability

The raw data of the present study are available from the corresponding author upon reasonable request.

## Supplementary Materials

Supplementary Materials The supplementary materials include additional data and analyses supporting the findings of the current study. They are: Table S1: channels and their corresponding brain regions. Table S2: pairwise comparison for reaction time and accuracy. Table S3: differences in Channel activation of deoxyhemoglobin (HbR) between the anxiety group and the health controls. Table S4: correlation analysis between all studied variables. Figure S1: reaction time and accuracy data for Stroop task.
