# Supplementary material for: Exploring Neural Correlates between Anxiety and Inhibitory Ability: Evidence from Task-Based fNIRS
Source: Depress Anxiety. 2024 Jun 27;2024:8680134. doi: 10.1155/2024/8680134 (PMC11918997; doi:10.1155/2024/8680134)
Supplement: Supplementary Materials — The supplementary materials include additional data and analyses supporting the findings of the current study. They are: Table S1: channels and their corresponding brain regions. Table S2: pairwise comparison for reaction time and accuracy. Table S3: differences in Channel activation of deoxyhemoglobin (HbR) between the anxiety group and the health controls. Table S4: correlation analysis between all studied variables. Figure S1: reaction time and accuracy data for Stroop task. [file 8680134.f1.docx]

**Supplementary Table 1**. The channels and the corresponding brain regions

| Label of SD or CH | Brodmann Area (Chris rorden' MRIcro) | Percentage |
| --- | --- | --- |
| S2 | 38 - Temporopolar area | 0.1761 |
|  | 45 - pars triangularis Broca's area | 0.2641 |
|  | 46 - Dorsolateral prefrontal cortex | 0.1831 |
|  | 47 - Inferior prefrontal gyrus | 0.3768 |
|  |  |  |
| S3 | 10 - Frontopolar area | 0.0468 |
|  | 11 - Orbitofrontal area | 0.9532 |
|  |  |  |
| S4 | 10 - Frontopolar area | 0.2226 |
|  | 11 - Orbitofrontal area | 0.6749 |
|  | 46 - Dorsolateral prefrontal cortex | 0.0177 |
|  | 47 - Inferior prefrontal gyrus | 0.0848 |
|  |  |  |
| S5 | 21 - Middle Temporal gyrus | 0.2583 |
|  | 38 - Temporopolar area | 0.7417 |
|  |  |  |
| S8 | 10 - Frontopolar area | 0.048 |
|  | 45 - pars triangularis Broca's area | 0.112 |
|  | 46 - Dorsolateral prefrontal cortex | 0.84 |
|  |  |  |
| S9 | 10 - Frontopolar area | 1 |
|  |  |  |
| S10 | 45 - pars triangularis Broca's area | 0.4409 |
|  | 46 - Dorsolateral prefrontal cortex | 0.5591 |
|  |  |  |
| D2 | 10 - Frontopolar area | 0.2605 |
|  | 11 - Orbitofrontal area | 0.021 |
|  | 46 - Dorsolateral prefrontal cortex | 0.3908 |
|  | 47 - Inferior prefrontal gyrus | 0.3277 |
|  |  |  |
| D3 | 10 - Frontopolar area | 0.5252 |
|  | 11 - Orbitofrontal area | 0.4748 |
|  |  |  |
| D4 | 46 - Dorsolateral prefrontal cortex | 0.8043 |
|  | 47 - Inferior prefrontal gyrus | 0.1957 |
|  |  |  |
| D7 | 44 - pars opercularis_ part of Broca's area | 0.0586 |
|  | 45 - pars triangularis Broca's area | 0.9414 |
|  |  |  |
| D8 | 10 - Frontopolar area | 0.9577 |
|  | 46 - Dorsolateral prefrontal cortex | 0.0423 |
|  |  |  |
| D9 | 10 - Frontopolar area | 0.6602 |
|  | 46 - Dorsolateral prefrontal cortex | 0.3398 |
|  |  |  |
| D10 | 6 - Pre-Motor and Supplementary Motor Cortex | 0.0621 |
|  | 44 - pars opercularis_ part of Broca's area | 0.531 |
|  | 45 - pars triangularis Broca's area | 0.4069 |
|  |  |  |
| CH1 (S2-D2) | 46 - Dorsolateral prefrontal cortex | 0.5305 |
|  | 47 - Inferior prefrontal gyrus | 0.4695 |
|  |  |  |
| CH2 (S2-D7) | 38 - Temporopolar area | 0.109 |
|  | 45 - pars triangularis Broca's area | 0.8462 |
|  | 46 - Dorsolateral prefrontal cortex | 0.0321 |
|  | 47 - Inferior prefrontal gyrus | 0.0128 |
|  |  |  |
| CH3 (S3-D2) | 10 - Frontopolar area | 0.2362 |
|  | 11 - Orbitofrontal area | 0.5203 |
|  | 46 - Dorsolateral prefrontal cortex | 0.0554 |
|  | 47 - Inferior prefrontal gyrus | 0.1882 |
|  |  |  |
| CH4 (S3-D3) | 10 - Frontopolar area | 0.2829 |
|  | 11 - Orbitofrontal area | 0.7171 |
|  |  |  |
| CH5 (S3-D8) | 10 - Frontopolar area | 0.755 |
|  | 11 - Orbitofrontal area | 0.245 |
|  |  |  |
| CH6 (S4-D3) | 10 - Frontopolar area | 0.1754 |
|  | 11 - Orbitofrontal area | 0.8246 |
|  |  |  |
| CH7 (S4-D4) | 10 - Frontopolar area | 0.3223 |
|  | 11 - Orbitofrontal area | 0.0379 |
|  | 46 - Dorsolateral prefrontal cortex | 0.3934 |
|  | 47 - Inferior prefrontal gyrus | 0.2464 |
|  |  |  |
| CH8 (S4-D9) | 10 - Frontopolar area | 0.8204 |
|  | 11 - Orbitofrontal area | 0.1796 |
|  |  |  |
| CH9 (S5-D4) | 38 - Temporopolar area | 0.0437 |
|  | 45 - pars triangularis Broca's area | 0.3452 |
|  | 46 - Dorsolateral prefrontal cortex | 0.2579 |
|  | 47 - Inferior prefrontal gyrus | 0.3532 |
|  |  |  |
| CH10 (S5-D10) | 6 - Pre-Motor and Supplementary Motor Cortex | 0.0403 |
|  | 38 - Temporopolar area | 0.1544 |
|  | 44 - pars opercularis_ part of Broca's area | 0.1409 |
|  | 45 - pars triangularis Broca's area | 0.3624 |
|  | 48 - Retrosubicular area | 0.302 |
|  |  |  |
| CH11 (S8-D2) | 10 - Frontopolar area | 0.3813 |
|  | 46 - Dorsolateral prefrontal cortex | 0.6187 |
|  |  |  |
| CH12 (S8-D7) | 45 - pars triangularis Broca's area | 0.8058 |
|  | 46 - Dorsolateral prefrontal cortex | 0.1942 |
|  |  |  |
| CH13 (S8-D8) | 10 - Frontopolar area | 0.4699 |
|  | 46 - Dorsolateral prefrontal cortex | 0.5301 |
|  |  |  |
| CH14 (S9-D3) | 10 - Frontopolar area | 1 |
|  |  |  |
| CH15 (S9-D8) | 10 - Frontopolar area | 1 |
|  |  |  |
| CH16 (S9-D9) | 10 - Frontopolar area | 1 |
|  |  |  |
| CH17 (S10-D4) | 45 - pars triangularis Broca's area | 0.1736 |
|  | 46 - Dorsolateral prefrontal cortex | 0.8264 |
|  |  |  |
| CH18 (S10-D9) | 10 - Frontopolar area | 0.1213 |
|  | 46 - Dorsolateral prefrontal cortex | 0.8787 |
|  |  |  |
| CH19 (S10-D10) | 45 - pars triangularis Broca's area | 1 |

***Note.*** S = source; D = detector; CH = Channel.

**Supplementary Table 2**. Pairwise comparison for reaction time and accuracy

| **Contrast** | **Estimate** | ***SE*** | ***t*** | ***p*** |
| --- | --- | --- | --- | --- |
| **Reaction time (RT)** |  |  |  |  |
| ANX Congruent - HCs Congruent | 0.290 | 0.022 | 13.046 | <0.001 |
| ANX Congruent - ANX Incongruent | -0.130 | 0.008 | -16.716 | <0.001 |
| ANX Congruent - HCs Incongruent | 0.227 | 0.025 | 8.977 | <0.001 |
| HCs Congruent - ANX Incongruent | -0.420 | 0.025 | -16.640 | <0.001 |
| HCs Congruent - HCs Incongruent | -0.063 | 0.008 | -8.113 | <0.001 |
| ANX Incongruent - HCs Incongruent | 0.357 | 0.028 | 12.769 | <0.001 |
| **Accuracy** |  |  |  |  |
| ANX Congruent - HCs Congruent | -0.074 | 0.028 | -2.651 | 0.055 |
| ANX Congruent - ANX Incongruent | 0.049 | 0.007 | 6.601 | <0.001 |
| ANX Congruent - HCs Incongruent | -0.069 | 0.029 | -2.327 | 0.130 |
| HCs Congruent - ANX Incongruent | 0.123 | 0.029 | 4.189 | <0.001 |
| HCs Congruent - HCs Incongruent | 0.005 | 0.007 | 0.717 | 1.000 |
| ANX Incongruent - HCs Incongruent | -0.118 | 0.031 | -3.813 | <0.05 |

***Note.*** ANX = Anxiety group.

**Supplementary Table 3**. Differences in channel activation (HbR) between groups

| CH | S-D | T | p |
| --- | --- | --- | --- |
| 1 | S2-D2 | 2.220 | 0.101 |
| 2 | S2-D7 | 2.169 | 0.101 |
| 3 | S3-D2 | 1.892 | 0.129 |
| 4 | S3-D3 | 2.813 | <0.05 |
| 5 | S3-D8 | 2.106 | 0.101 |
| 6 | S4-D3 | 0.470 | 0.715 |
| 7 | S4-D4 | 3.856 | <0.05 |
| 8 | S4-D9 | 0.189 | 0.850 |
| 9 | S5-D4 | 2.380 | 0.090 |
| 10 | S5-D10 | 0.390 | 0.736 |
| 11 | S8-D2 | 1.462 | 0.214 |
| 12 | S8-D7 | 1.778 | 0.148 |
| 13 | S8-D8 | 0.746 | 0.579 |
| 14 | S9-D3 | 1.681 | 0.159 |
| 15 | S9-D8 | 0.897 | 0.504 |
| 16 | S9-D9 | -1.656 | 0.159 |
| 17 | S10-D4 | 4.033 | <0.05 |
| 18 | S10-D9 | 0.498 | 0.715 |
| 19 | S10-D10 | 2.027 | 0.107 |

***Note.*** S = Source; D = Detector; CH = Channel

**Supplementary Table 4.** Correlation analysis between all studied variables (*n* = 118)

|  | **SAS** | **ACC Differ** | **RT Differ** | **CH1** | **CH2** | **CH3** | **CH4** | **CH5** | **CH6** | **CH7** | **CH8** | **CH9** | **CH10** | **CH11** | **CH12** | **CH13** | **CH14** | **CH15** | **CH16** | **CH17** | **CH18** | **CH19** |
| --- | --- | --- | --- | --- | --- | --- | --- | --- | --- | --- | --- | --- | --- | --- | --- | --- | --- | --- | --- | --- | --- | --- |
| **SAS** | 1 |  |  |  |  |  |  |  |  |  |  |  |  |  |  |  |  |  |  |  |  |  |
| **ACC Differ** | .46*** | 1 |  |  |  |  |  |  |  |  |  |  |  |  |  |  |  |  |  |  |  |  |
| **RT Differ** | .73*** | .44*** | 1 |  |  |  |  |  |  |  |  |  |  |  |  |  |  |  |  |  |  |  |
| **CH1** | -.31** | -.14 | -.26** | 1 |  |  |  |  |  |  |  |  |  |  |  |  |  |  |  |  |  |  |
| **CH2** | -.35*** | -.16 | -.39*** | .60*** | 1 |  |  |  |  |  |  |  |  |  |  |  |  |  |  |  |  |  |
| **CH3** | -.24* | -.12 | -.21* | .44*** | .49*** | 1 |  |  |  |  |  |  |  |  |  |  |  |  |  |  |  |  |
| **CH4** | -.25* | -.18 | -.29** | .32*** | .59*** | .23* | 1 |  |  |  |  |  |  |  |  |  |  |  |  |  |  |  |
| **CH5** | -.34*** | -.16 | -.36*** | .43*** | .49*** | .46*** | .32*** | 1 |  |  |  |  |  |  |  |  |  |  |  |  |  |  |
| **CH6** | -.17 | -.05 | -.23* | .08 | .05 | .30** | .09 | .31** | 1 |  |  |  |  |  |  |  |  |  |  |  |  |  |
| **CH7** | -.26** | -.05 | -.24* | .58*** | .43*** | .19* | .23* | .12 | .01 | 1 |  |  |  |  |  |  |  |  |  |  |  |  |
| **CH8** | -.20* | .01 | -.24* | .23* | .26** | .33*** | .20* | .51*** | .25* | .09 | 1 |  |  |  |  |  |  |  |  |  |  |  |
| **CH9** | -.23* | -.11 | -.21* | .51*** | .51*** | .33*** | .24* | .43*** | .03 | .40*** | .24* | 1 |  |  |  |  |  |  |  |  |  |  |
| **CH10** | -.22* | -.09 | -.28** | .48*** | .55*** | .42*** | .43*** | .48*** | .20* | .22* | .30** | .33*** | 1 |  |  |  |  |  |  |  |  |  |
| **CH11** | -.33*** | -.07 | -.40*** | .56*** | .65*** | .56*** | .42*** | .60*** | .19* | .23* | .45*** | .56*** | .58*** | 1 |  |  |  |  |  |  |  |  |
| **CH12** | -.29** | -.19 | -.31** | .61*** | .52*** | .33*** | .14 | .28** | .02 | .54*** | .09 | .51*** | .32*** | .39*** | 1 |  |  |  |  |  |  |  |
| **CH13** | -.35*** | -.21* | -.41*** | .41*** | .53*** | .46*** | .40*** | .72*** | .14 | .15 | .45*** | .38*** | .51*** | .66*** | .25* | 1 |  |  |  |  |  |  |
| **CH14** | -.07 | -.02 | -.12 | .14 | .14 | .16 | .05 | .33*** | .30** | .10 | .28** | .20* | .17 | .27** | .12 | .29** | 1 |  |  |  |  |  |
| **CH15** | -.51*** | -.18 | -.45*** | .51*** | .63*** | .42*** | .37*** | .44*** | .31** | .45*** | .31** | .53*** | .46*** | .56*** | .47*** | .42*** | .10 | 1 |  |  |  |  |
| **CH16** | -.26** | -.22* | -.34*** | .24* | .42*** | .19* | .37*** | .37*** | .26** | .22* | .31** | .26** | .35*** | .42*** | .17 | .36*** | .28** | .45*** | 1 |  |  |  |
| **CH17** | -.47*** | -.26** | -.68*** | .38*** | .47*** | .36*** | .35*** | .52*** | .28** | .23* | .42*** | .44*** | .56*** | .59*** | .39*** | .49*** | .21* | .51*** | .41*** | 1 |  |  |
| **CH18** | -.25** | -.10 | -.36*** | .55*** | .71*** | .51*** | .42*** | .68*** | .09 | .23* | .38*** | .43*** | .63*** | .78*** | .36*** | .63*** | .25** | .45*** | .38*** | .56*** | 1 |  |
| **CH19** | -.23* | -.10 | -.26** | .47*** | .37*** | .18 | .10 | .28** | 0.001 | .44*** | .14 | .43*** | .39*** | .28** | .57*** | .27** | .08 | .47*** | .21* | .41*** | .27** | 1 |
| **VFT** | -.78*** | -.35*** | -.59*** | .30** | .28** | .21* | .18 | .29** | .17 | .25* | .21* | .23* | .19* | .31** | .23* | .27** | .13 | .46*** | .22* | .50*** | .23* | .23* |

***Note.*** SAS = the score of Self-rating Anxiety Scale; RT Differ = difference time between incongruent and congruent condition; ACC Differ = difference accuracy between incongruent and congruent condition; CH = Channel; VFT = verbal fluency task performance; * = *p* < 0.05, ** = *p* < 0.01, *** = *p* < 0.001


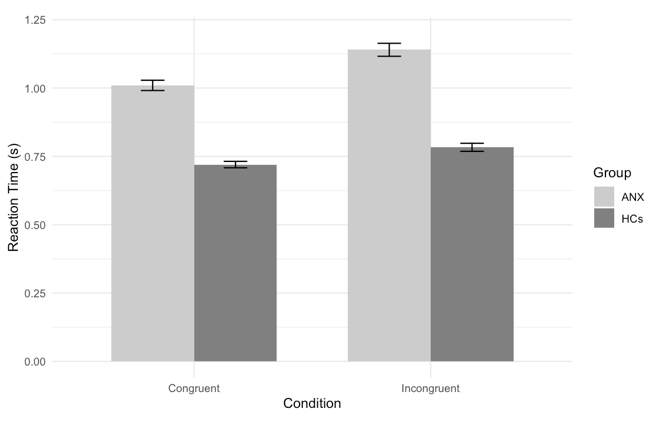

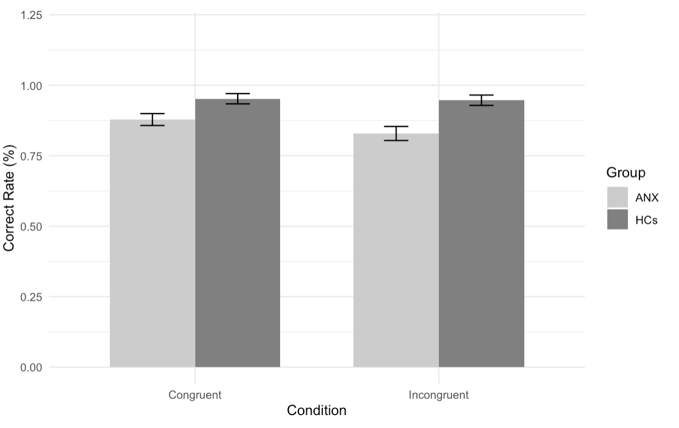


**Supplementary Fig. 1.** Reaction time and accuracy data for Stroop task.

(left) Mean reaction time (RT) in seconds (s) and (right) accuracy. ANX stands for anxiety group.
